# APACHE II and NUTRIC Scores for Mortality Prediction in Chronic Critical Illness: A “Right-Side” Prognostic Modeling Approach

**DOI:** 10.3390/diagnostics15243218

**Published:** 2025-12-16

**Authors:** Dmitrij V. Zhidilyaev, Levan B. Berikashvili, Mikhail Ya. Yadgarov, Petr A. Polyakov, Alexey A. Yakovlev, Artem N. Kuzovlev, Valery V. Likhvantsev

**Affiliations:** 1 Moscow City Health Department, State Budgetary Institution Konchalovsky City Clinical Hospital, 124489 Moscow, Russia; zhidilyaevdv@zdrav.mos.ru; 2 Federal Research and Clinical Center of Intensive Care Medicine and Rehabilitology, 107031 Moscow, Russia; lberikashvili@fnkcrr.ru (L.B.B.); p.polyakov@fnkcrr.ru (P.A.P.); ayakovlev@fnkcrr.ru (A.A.Y.); artem_kuzovlev@fnkcrr.ru (A.N.K.); lik0704@gmail.com (V.V.L.)

**Keywords:** chronic critical illness, prognosis, APACHE II, NUTRIC, mortality, open data set

## Abstract

**Background/Objectives:** Accurate prognostication for patients with chronic critical illness (CCI) following brain injury remains challenging. Conventional scoring systems like the Acute Physiology and Chronic Health Evaluation II (APACHE II) and the Nutrition Risk in the Critically Ill (NUTRIC) score are validated as “left-side” models for risk stratification at intensive care unit (ICU) admission but may not capture the evolving trajectory of prolonged illness. This study aimed to evaluate the prognostic performance of APACHE II and NUTRIC as “right-side” models—assessed at intervals closer to the outcome—by testing the hypothesis that their predictive accuracy for in-hospital mortality improves when measured nearer to the time of death. **Methods:** In this real-world data analysis study, data were extracted from the electronic health records (Russian Intensive Care Dataset [RICD] v. 2.0) of 328 adult patients with CCI following brain injury. The discriminative ability of repeatedly assessed APACHE II and NUTRIC scores for predicting mortality was analyzed by calculating the area under the receiver operating characteristic curve (AUROC) for three predefined intervals before death: within ≤7 days, 8–14 days, and ≥15 days. **Results:** Among the 328 patients (median age 64 years; 18.3% in-hospital mortality), a total of 380 paired score assessments were analyzed. The predictive performance for both scores was highest within 7 days of death (APACHE II AUROC: 0.883; NUTRIC AUROC: 0.839). Discriminatory ability declined at 8–14 days (APACHE II AUROC: 0.807; NUTRIC AUROC: 0.778) and was poorest at ≥15 days before death (APACHE II AUROC: 0.671; NUTRIC AUROC: 0.681). The NUTRIC score consistently demonstrated higher AUROC values than APACHE II across all intervals, though the differences were not statistically significant. **Conclusions:** In patients with CCI following brain injury, the prognostic accuracy of APACHE II and NUTRIC scores is time-dependent, peaking immediately before death and offering poor long-term prediction from admission. These findings underscore the limitation of static, admission-based models and highlight the necessity for developing dynamic, personalized and time-sensitive prognostic tools tailored to the evolving course of chronic critical illness.

## 1. Introduction

Chronic Critical Illness (CCI) is recognized as a distinct nosological entity, characterized by the persistence of profound multi-organ dysfunction necessitating sustained intensive care interventions, irrespective of the resolution of the inciting acute pathology [1,2,3]. The syndrome is associated with a markedly unfavorable prognostic trajectory. Epidemiological data indicate an approximate in-hospital mortality of 27%, with cumulative mortality rising to 45% at one-year post-identification [4]. Long-term survivors are predominantly characterized by severe and persistent functional dependency, typically mandating institutionalization in long-term acute care or skilled nursing facilities, concomitant with a significant and sustained deterioration in health-related quality of life metrics [4,5,6,7,8]. A clinically significant etiological subgroup within the CCI cohort consists of patients with severe traumatic brain injury (TBI), representing 6.2% of the diagnosed population.

Critically ill patients with prolonged or chronic critical illness following brain injury represent one of the most resource-intensive groups in intensive care units (ICUs). These patients often experience complex pathophysiological changes, including persistent organ dysfunction, impaired neurological recovery, and a high risk of adverse outcomes, particularly death [9,10,11]. Accurate prognostication in this population remains challenging. Conventional scoring systems, although widely used, were primarily designed for risk stratification at admission and may fail to capture the evolving trajectory of chronic critical illness [12,13,14].

Among the most established tools, the Acute Physiology and Chronic Health Evaluation II (APACHE II) has been validated across heterogeneous ICU populations as an admission-based predictor of mortality [12,15]. Similarly, the Nutrition Risk in the Critically Ill (NUTRIC) score was developed to identify patients at nutritional risk at the time of ICU admission, incorporating both clinical and inflammatory markers [16,17,18]. Both instruments, however, were conceived as “left-side” prognostic models—intended for use at the start of ICU stay—and not for repeated evaluation during long-term critical illness.

For patients with chronic critical illness, the prognostic accuracy of admission-based models is limited. Once survival beyond the acute phase is achieved, the predictive power of left-sided models declines substantially, as these patients follow highly variable trajectories influenced by complications, comorbidities, and prolonged organ support [19,20,21]. In this context, the concept of “right-side” prognostic modeling—evaluating scores in proximity to the outcome rather than at admission—may offer greater clinical relevance. By repeatedly applying established scores at later stages of ICU stay, it becomes possible to estimate the likelihood of survival or death more accurately in patients whose illness course extends far beyond the initial resuscitation period.

To our knowledge, no previous studies have systematically evaluated the prognostic performance of APACHE II and NUTRIC scores as right-side models in patients with chronic critical illness following brain injury. We therefore aimed to assess their discriminative ability when calculated at different time intervals prior to death, hypothesizing that their predictive accuracy would improve when measured closer to the fatal outcome.

## 2. Materials and Methods

### 2.1. Data Sources

This real-world data analysis was conducted using data extracted from the electronic health records (EHRs) of the Federal Research and Clinical Center of Intensive Care Medicine and Rehabilitology (Russian Intensive Care Dataset, RICD v. 2.0 [22]). RICD includes comprehensive clinical and administrative information for all patients admitted to the center’s intensive care units (ICUs) between December 2017 and September 2024. All data were fully deidentified prior to analysis. In accordance with institutional policy and national regulations, the local ethics committee confirmed that formal ethical approval was not required for this analysis of anonymized data.

The study was reported in accordance with the Strengthening the Reporting of Observational Studies in Epidemiology (STROBE) guidelines [23]. A completed STROBE checklist is provided in Table S1 (Supplementary Materials).

### 2.2. Selection Criteria and Study Population

All patients admitted to the Federal Research and Clinical Center of Intensive Care Medicine and Rehabilitology with prolonged or chronic critical illness following brain injury were screened for eligibility. Patients were included if they had at least one documented assessment using both the Acute Physiology and Chronic Health Evaluation II (APACHE II) and the Nutrition Risk in the Critically Ill (NUTRIC) scores during their ICU stay. Repeated ICU admissions were excluded to ensure independent observations.

### 2.3. Data Extraction

Data were extracted from the electronic health records using SQLite version 3.46.1 (https://www.sqlite.org/) and DB Browser for SQLite version 3.13.1. The following parameters were analyzed: demographic data (sex, age, body mass index [BMI]); clinical characteristics, including scores at ICU admission–APACHE II, NUTRIC, Sequential Organ Failure Assessment (SOFA), Full Outline of UnResponsiveness (FOUR), Glasgow Coma Scale (GCS), Coma Recovery Scale—Revised (CRS-R), the Disability Rating Scale (DRS) and relevant comorbidities; outcome variables including hospital mortality, length of stay in the ICU and hospital, need for mechanical ventilation (MV), and administration of vasoactive medications. In cases where APACHE II scores were not available in the medical record, they were calculated retrospectively based on current guidelines, with a frequency of one assessment per week starting from the first day of ICU admission. The NUTRIC score was subsequently assessed at the same time points.

### 2.4. Outcomes

The primary outcome of the study was the area under the receiver operating characteristic curve (AUROC). Discriminative performance of the APACHE II and NUTRIC scores for predicting in-hospital mortality was assessed across different time intervals prior to death. Specifically, AUROC values were calculated for score assessments performed within three predefined intervals preceding the fatal outcome: within ≤7 days, 8–14 days, and ≥15 days before death.

### 2.5. Statistical Analysis

All statistical analyses were performed using IBM SPSS Statistics for Windows version 29.0 (IBM Corp., Armonk, NY, USA) and MedCalc^®^ Statistical Software version 20.305 (MedCalc Software Ltd., Ostend, Belgium; https://www.medcalc.org; 2023). Continuous variables were expressed as medians with interquartile ranges (IQRs), and categorical variables as absolute frequencies and percentages. Data distribution was assessed using the Shapiro–Wilk test and visual inspection of histograms.

Between-group comparisons of continuous variables were performed using the Mann–Whitney U test. Categorical variables were compared using the χ^2^ test or Fisher exact test, as appropriate. The area under the receiver operating characteristic curve (AUROC) was estimated using a non-parametric approach with calculation of 95% confidence intervals. Comparisons of AUROC values between the APACHE II and NUTRIC scores were performed using the DeLong et al. (1988) method [24]. Optimal cutoff points were determined using the Youden index, defined as the maximum of (sensitivity + specificity − 1). Sensitivity and specificity were calculated at the corresponding optimal cutoff.

All statistical tests were two-sided, and a *p*-value < 0.05 was considered to indicate statistical significance.

## 3. Results

### 3.1. Patient Characteristics

**Figure 1:**
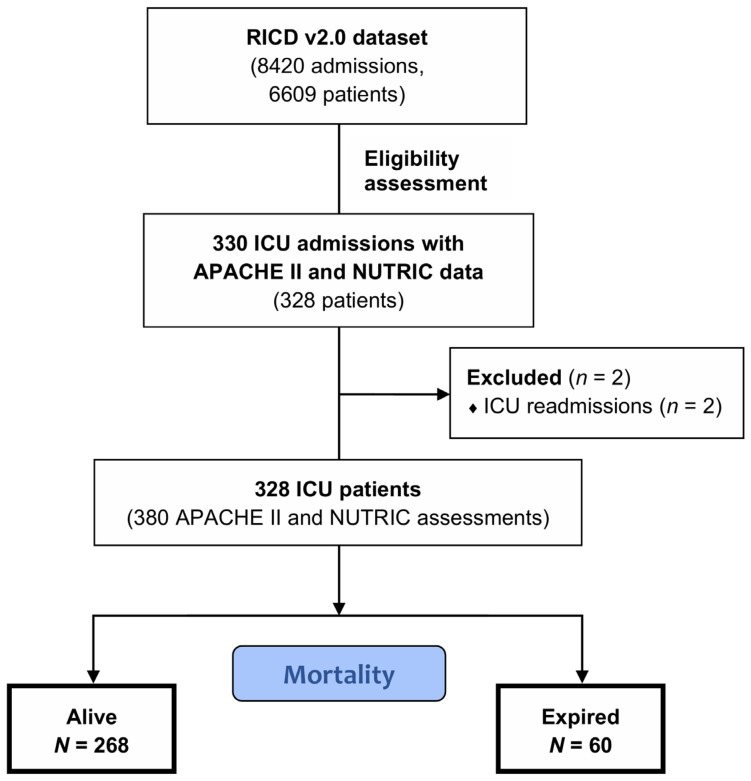
Flowchart of patient selection in the study.

A total of 330 ICU admissions were screened for eligibility, corresponding to 328 unique patients. After exclusion of 2 repeated ICU admissions, 328 patients (167 men; median age, 64 years [interquartile range (IQR), 48–75 years]) were included in the final analysis. The patient selection process is illustrated in Figure 1.

Baseline characteristics, clinical scores at ICU admission, and comorbidities stratified by survival status are presented in Table 1. The median length of hospital stay was 49 days (IQR, 22–63), and the median ICU stay was 29 days (IQR, 19–51). In-hospital mortality occurred in 60 patients (18.3%). MV was used in 24 patients (7.3%), and vasoactive agents were administered to 131 patients (41.8%).

A total of 380 paired assessments of APACHE II and NUTRIC scores were available. Compared with survivors, non-survivors were older and had higher scores on the APACHE II, NUTRIC, SOFA, FOUR, GCS, and DRS scales at admission. Additionally, non-survivors more frequently had type 2 diabetes mellitus and chronic kidney disease (Table 1).

### 3.2. Effectiveness of Scales

The prognostic performance of the APACHE II and NUTRIC scores for in-hospital mortality was highest when assessed within 7 days prior to death, with AUROC values of 0.883 and 0.839, respectively. Between 8 and 14 days prior to death, discriminatory ability declined, with AUROC values of 0.807 for APACHE II and 0.778 for NUTRIC. When assessed ≥15 days before death, the predictive performance further decreased, with AUROC values of 0.671 and 0.681, respectively, accompanied by unacceptably low sensitivity.

**Figure 2:**
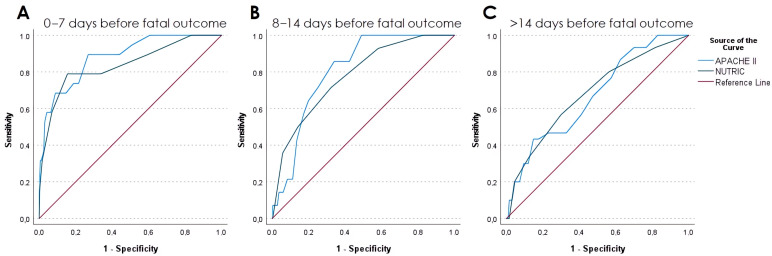
Predictive value of APACHE II score and NUTRIC score in ICU patients within three predefined intervals preceding the fatal outcome.

Across all time intervals, the AUROC values for APACHE II and NUTRIC were statistically comparable (*p* > 0.05; Table 2, Figure 2).

## 4. Discussion

### 4.1. Key Findings

This study was designed to test the hypothesis that the predictive accuracy of admission-based severity scores (APACHE II and NUTRIC) for in-hospital mortality would improve when these scores were recalculated closer to the fatal outcome in patients with chronic critical illness. In line with this objective, our key findings confirm this hypothesis. We demonstrated that neither APACHE II nor NUTRIC provided stable prognostic value throughout the ICU course. Instead, their discriminative ability was temporally dynamic, peaking in the week immediately preceding death and progressively diminishing when applied to earlier time points, including admission. Although NUTRIC consistently outperformed APACHE II, both models showed a limited capacity to forecast outcomes from baseline data alone in this CCI population.

### 4.2. Relationship with Previous Studies

Our results extend previous observations regarding the diminishing prognostic value of admission-based severity scores in prolonged ICU stays [9,25,26]. While prior research focused primarily on APACHE-derived models, our findings add important nuance by directly comparing APACHE II with NUTRIC in a CCI population. Our recent evidence highlights the relevance of nutritional and inflammatory status in long-term outcomes of critical illness [27], supporting the superior persistence of NUTRIC performance compared to APACHE II in our cohort. Moreover, contemporary studies emphasize the need for dynamic risk assessment strategies that adapt to evolving patient trajectories rather than relying on a single baseline measure [28,29].

### 4.3. Significance of the Study Findings

These findings suggest that the clinical trajectory of CCI is driven more by evolving physiologic and metabolic changes than by the initial severity at admission. Although both APACHE II and NUTRIC scores capture outcome-relevant factors, their discriminative power tends to increase only as patients approach the terminal phase, which limits their usefulness for early, long-term prognostication. This reflects an obvious reality: the greater the time gap between admission and outcome, the harder it is to accurately predict the outcome based on early data. Ongoing changes in clinical status and the effects of treatment strategies further confound prognostic accuracy. These observations highlight the need for dynamic models capable of delivering reliable personalized predictions earlier in the ICU course, when therapeutic decision-making and communication with families are most crucial.

### 4.4. Strengths and Limitations

The strengths of our study include the exclusive focus on a large population of CCI patients, systematic comparison of APACHE II and NUTRIC, and application of robust statistical methods to evaluate changes in predictive accuracy over time. Furthermore, the study design (an analysis of real-world clinical practice data) ensures high external and internal validity of the findings.

However, several limitations should be acknowledged. First, the single-center design may affect the generalizability of the findings to other patient populations and clinical settings. Second, we relied on routinely collected clinical data, which may be subject to documentation errors or missingness. Finally, external validity across different healthcare systems remains uncertain, as prognostic performance can vary depending on ICU organization and patient mix.

### 4.5. Further Investigations

Future research should aim to develop and validate prognostic models tailored specifically to CCI. In particular, dynamic approaches that incorporate evolving nutritional, metabolic, and inflammatory parameters may capture outcome trajectories more effectively than admission-based models. Multicenter, prospective studies are essential to confirm the superiority of such approaches across diverse healthcare settings. Importantly, future investigations should also consider patient-centered outcomes such as long-term functional recovery and quality of life, which are particularly relevant in the CCI population [30,31].

## 5. Conclusions

In patients with chronic critical illness, APACHE II and NUTRIC demonstrated their strongest predictive value close to death but were poor predictors at admission. NUTRIC consistently outperformed APACHE II, yet neither model offered reliable long-term prognostication. These findings highlight the urgent need for dynamic, time-sensitive models tailored to the evolving course of CCI.

## Figures and Tables

**Table 1:** Initial parameters, scales and comorbidities of ICU patients by outcome (mortality).

Parameters		Alive *N* = 268	Expired *N* = 60	*p*-Value
Sex	male	140, 52.2%	27, 45.0%	0.3 ^1^
	female	128, 47.8%	33, 55.0%	
Age, years		62 (IQR 48–74)	73 (IQR 56–82)	<0.001 ^2^
BMI, kg/m^2^		*N* = 241, 25 (IQR 23–29)	*N* = 56, 26 (IQR 23–30)	0.8 ^2^
Scales at admission				
APACHE II at admission, score		*N* = 225, 15 (IQR 11–18)	*N* = 54, 21 (IQR 16–27)	<0.001 ^2^
NUTRIC at admission, score		*N* = 225, 4 (IQR 3–5)	*N* = 54, 5 (IQR 4–7)	<0.001 ^2^
SOFA at admission, score		*N* = 265, 4 (IQR 3–5)	*N* = 58, 5 (IQR 3–7)	<0.001 ^2^
FOUR at admission, score		*N* = 257, 13 (IQR 11–16)	*N* = 55, 12 (IQR 8–14)	0.016 ^2^
GCS at admission, score		*N* = 263, 11 (IQR 9–13)	*N* = 58, 10 (IQR 7–12)	0.029 ^2^
CRS-R at admission, score		*N* = 128, 13 (IQR 6–20)	*N* = 22, 8 (IQR 5–13)	0.067 ^2^
DRS at admission, score		*N* = 253, 21 (IQR 18–23)	*N* = 55, 23 (IQR 19–25)	0.027 ^2^
Comorbidity				
Ischemic stroke		130, 48.5%	36, 60.0%	0.1 ^1^
Hemorrhagic stroke		55, 20.5%	10, 16.7%	0.6 ^1^
Traumatic brain injury		43, 16.0%	9, 15.0%	0.9 ^1^
Anemia		62, 23.1%	14, 23.3%	0.9 ^1^
Type 2 diabetes mellitus		40, 14.9%	17, 28.3%	0.016 ^1^
Cerebrovascular disease		19, 7.1%	7, 11.7%	0.3 ^1^
Chronic kidney disease		31, 11.6%	17, 28.3%	0.002 ^1^
COPD		159, 59.3%	43, 71.7%	0.08 ^1^
Myocardial infarction		2, 0.7%	1, 1.7%	0.5 ^3^
Coronary artery disease		159, 59.3%	43, 71.7%	0.08 ^1^
Atrial fibrillation		30, 11.2%	12, 20.0%	0.09 ^1^
Arterial hypertension		223, 83.2%	50, 83.3%	0.9 ^1^
Coagulopathy		4, 1.5%	2, 3.3%	0.3 ^3^
Heart failure		56, 20.9%	12, 20.0%	0.9 ^1^
Polytrauma		12, 4.5%	1, 1.7%	0.5 ^3^
Malignant tumor		4, 1.5%	0, 0.0%	0.9 ^3^

Abbreviations: APACHE II, Acute Physiology and Chronic Health Evaluation II; COPD, chronic obstructive pulmonary disease; CRS-R, Coma Recovery Scale—Revised; DRS, Disability Rating Scale; FOUR, Full Outline of UnResponsiveness; GCS, Glasgow Coma Scale; ICU, intensive care unit; IQR, interquartile range; NUTRIC, Nutrition Risk in the Critically Ill. ^1^—chi-square test; ^2^—Mann–Whitney U test; ^3^—Fisher’s exact test.

**Table 2:** ROC-analysis of APACHE II and NUTRIC for mortality in ICU patients.

Predictive Variables	AUROC	95% CI	*p*-Value	Best Cutoff Value	Sensitivity	Specificity
0–7 days before fatal outcome						
APACHE II (19 pos. 361 neg.)	0.883	0.808–0.958	<0.001	19	89.5	73.1
NUTRIC (19 pos. 361 neg.)	0.839	0.732–0.946	<0.001	6	78.9	84.5
*p*-value *	0.245					
8–14 days before fatal outcome						
APACHE II (14 pos. 347 neg.)	0.807	0.730–0.884	<0.001	18	85.7	66.0
NUTRIC (14 pos. 347 neg.)	0.778	0.663–0.893	<0.001	5	71.4	67.7
*p*-value *	0.559					
≥15 days before fatal outcome						
APACHE II (30 pos. 317 neg.)	0.671	0.575–0.767	<0.001	21	43.3	85.2
NUTRIC (30 pos. 317 neg.)	0.681	0.579–0.782	<0.001	5	56.7	70.0
*p*-value *	0.748					

Abbreviations: APACHE II, Acute Physiology and Chronic Health Evaluation II; AUROC, Area Under the Receiver Operating Characteristic curve; CI, confidence interval; NUTRIC, Nutrition Risk in the Critically Ill. * DeLong et al., 1988 [24].

## Data Availability

Restrictions apply to the availability of these data. Data were obtained from RICD and are available at https://fnkcrr-database.ru (accessed on 20 June 2024) with the permission of Federal Research and Clinical Center of Intensive Care Medicine and Rehabilitology.

## Supplementary Materials

The following supporting information can be downloaded at: https://www.mdpi.com/article/10.3390/diagnostics15243218/s1, Table S1: STROBE Checklist.

## Abbreviations

The following abbreviations are used in this manuscript:

CCI	chronic critical illness
APACHE II	Acute Physiology and Chronic Health Evaluation II
NUTRIC	Nutrition Risk in the Critically Ill
AUROC	area under the receiver operating characteristic curve
ICU	intensive care unit
EHR	electronic health record
RICD	Russian Intensive Care Dataset
BMI	body mass index
SOFA	Sequential Organ Failure Assessment
FOUR	Full Outline of UnResponsiveness
GCS	Glasgow Coma Scale
CRS-R	Coma Recovery Scale—Revised
DRS	Disability Rating Scale
MV	mechanical ventilation
IQR	interquartile range
ROC	receiver operating characteristic
COPD	chronic obstructive pulmonary disease
CI	confidence interval
